# Clinical relevance of circulating tumor DNA in ovarian cancer: current issues and future opportunities

**DOI:** 10.37349/etat.2024.00239

**Published:** 2024-06-19

**Authors:** Elena Trevisi, Cristiana Sessa, Ilaria Colombo

**Affiliations:** IRCCS Istituto Romagnolo per lo Studio dei Tumori (IRST) "Dino Amadori", Italy; Oncology Institute of Southern Switzerland (IOSI), Ente Ospedaliero Cantonale (EOC), 6500 Bellinzona, Switzerland

**Keywords:** Liquid biopsy, ovarian cancer, circulating tumor DNA, cell-free DNA

## Abstract

Ovarian cancer (OC) is the most lethal gynecologic malignancy worldwide. Due to the lack of effective screening and early detection strategies, many patients with OC are diagnosed with advanced disease, where treatment is rarely curative. Moreover, OC is characterized by high intratumor heterogeneity, which represents a major barrier to the development of effective treatments. Conventional tumor biopsy and blood-based biomarkers, such as cancer antigen 125 (CA125), have different limitations. Liquid biopsy has recently emerged as an attractive and promising area of investigation in oncology, due to its minimally invasive, safe, comprehensive, and real-time dynamic nature. Preliminary evidence suggests a potential role of liquid biopsy to refine OC management, by improving screening, early diagnosis, assessment of response to treatment, detection, and profiling of drug resistance. The current knowledge and the potential clinical value of liquid biopsy in OC is discussed in this review to provide an overview of the clinical settings in which its use might support and improve diagnosis and treatment.

## Introduction

Ovarian cancer (OC) is the fifth most common cause of cancer mortality in women worldwide and the main cause of cancer-related death for gynecological malignancies [1]. Among the different histological subtypes, high-grade serous OC (HGSOC) is the most prevalent [2]. Unfortunately, due to the lack of an effective screening strategy and specific symptoms, the majority of patients are diagnosed with advanced disease [3]. Advanced-stage OC is managed with debulking surgery with the aim of optimal cytoreduction and platinum-based systemic therapy administered after surgery or in the neoadjuvant setting [2–5]. More recently, the use of the antiangiogenic agent bevacizumab and poly-ADP ribose polymerase (PARP) inhibitors as maintenance in the front line has been shown to improve patients’ outcomes [6–8]. However, despite the initial chemosensitivity, many patients relapse and ultimately develop platinum resistance. This is strictly related both to the high intratumoral heterogeneity in the primary tumor and to the spatial-temporal genomic evolution under the selective pressure of systemic treatments.

Understanding the complex genomic background of OC could guide the use of targeted therapies, which will pave the way for the implementation of precision medicine. To date, the only molecular characteristics routinely used for decision-making are breast cancer gene 1 and 2 (*BRCA1/2*) mutations and homologous recombination (HR) deficiency, which have been demonstrated and validated as predictive biomarkers of response to platinum therapy and PARP inhibitors in the frontline setting [7–10]. Thus, there is an urgent need to identify disease-specific biomarkers, which could improve the detection rate of OC and be implemented in treatment algorithms.

## Liquid biopsy as a new tool in oncology

**Figure 1:**
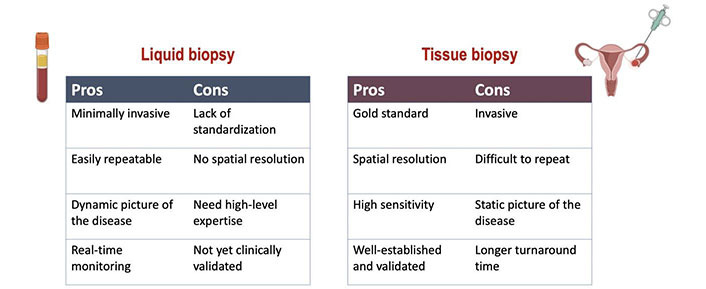
Comparison of the advantages and limitations of liquid versus tissue biopsy

The use of prognostic and predictive biomarkers to inform clinical decisions has become increasingly important and the majority are based on tumor tissue analysis. However, although it represents the gold standard for tumor molecular sequencing, tissue biopsy is associated with technical and safety issues. Indeed, it is an invasive procedure that may cause discomfort as well as complications for the patient, and it is not always feasible [11]. Moreover, cancer is a dynamic process characterized by space-time heterogeneity and evolution, which are often not fully captured by tumor biopsy samples [12–14]. These limitations highlight the need for more innovative and comprehensive methods to decipher the complexity of cancer. The most promising alternative to traditional tissue biopsy is the so-called “liquid biopsy”, a procedure involving the study of biological fluids such as blood, urine, saliva, stool, ascites, etc. from which cancer material can be isolated [15–17]. Compared with tissue-based sampling liquid biopsy is easier, safer, and definitely more convenient for both patients and health providers (Figure 1). Furthermore, liquid biopsy can be repeated over time due to its minimally invasive nature, thus making easier “real-time” disease monitoring [15]. Liquid biopsy based on the analysis of circulating tumor DNA (ctDNA) is currently the most attractive and investigated tool. ctDNA is the fraction of cell-free DNA (cfDNA) that is released from the tumor in the plasma. ctDNA is shed by the tumor cells as a result of apoptosis, necrosis, and active secretion, and it can be detected and quantified in cfDNA by tumor-specific genetic alterations [18].

Two different approaches were developed to investigate ctDNA. The first strategy involves querying a single or few tumor-specific mutations known from the primary tumor (“targeted strategy”). This approach is based on the use of polymerase chain reaction (PCR)-based assays, next generation sequencing (NGS)-based methods or Safe-sequencing system (Safe-SeqS), cancer personalized profile (CAPP-Seq), tagged amplicon deep sequencings (Tam-Seq). PCR-based methods detect a limited number of known mutations, while NGS can identify a broad spectrum of somatic aberrations, including single nucleotide variants (SNVs), copy number variations (CNVs), and chromosomal rearrangements. Overall, this strategy requires prior detailed knowledge of the tumor genome, but it is very sensitive and specific [19, 20]. The second approach to investigate ctDNA is through an “untargeted strategy” using whole exome sequencing (WES) or whole genome sequencing (WGS)*.* This approach has the advantage of being independent of the mutational profile of the tumor tissue and can identify novel alterations occurring during treatment, but has the disadvantage of being more expensive and less sensitive [19, 20].

Given the limitations of tissue-based analysis, liquid biopsy has been actively pursued as a potential tool to refine cancer patients’ care [21, 22]. We provide an overview of the potential role of liquid biopsy in OC.

## Potential roles of liquid biopsy in OC

**Figure 2:**
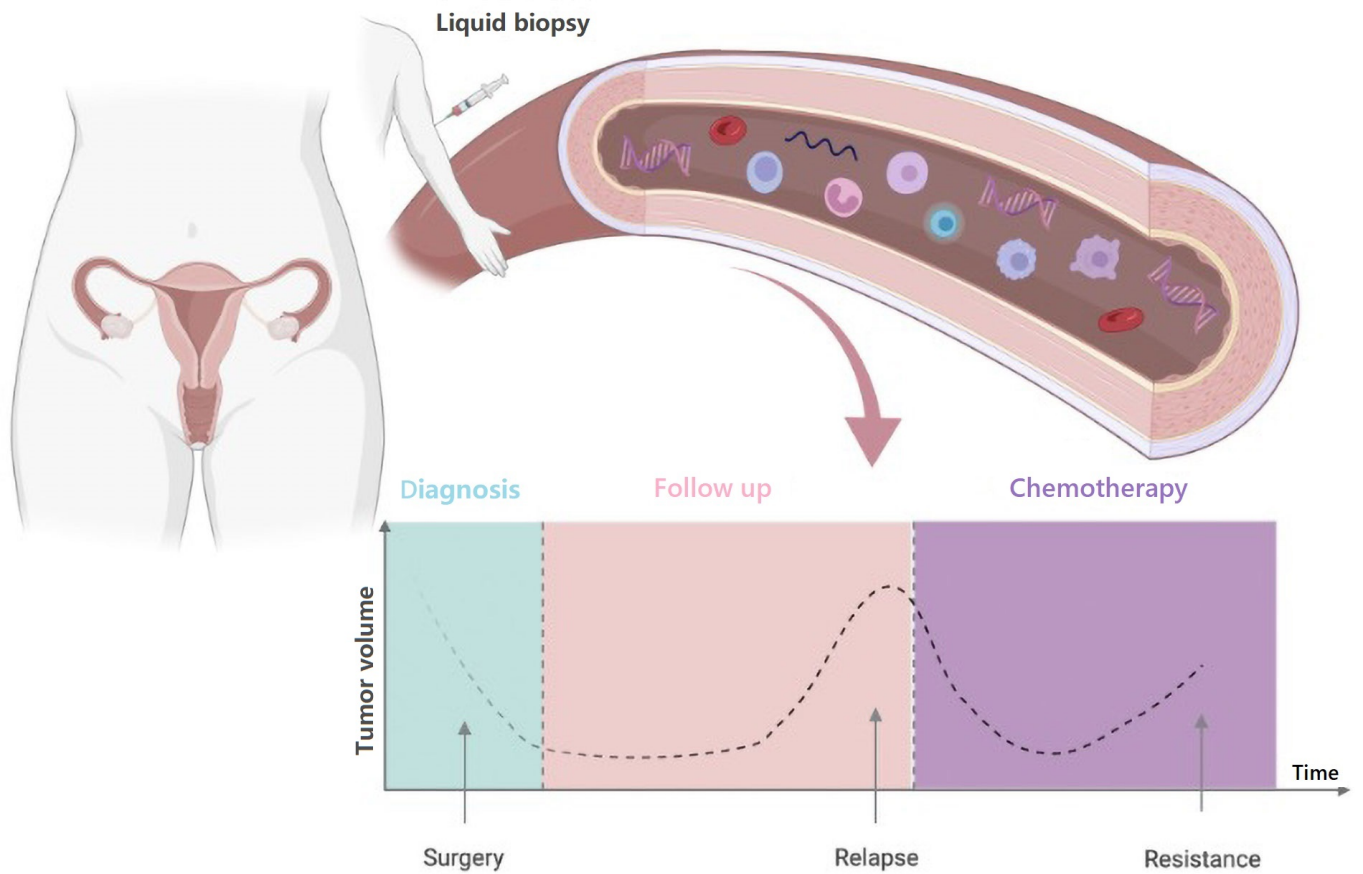
Timelines of potential clinical applications of blood-based liquid biopsy in OC. Created with BioRender.com

Potential applications of liquid biopsy include, among others, cancer screening and early diagnosis, detection of minimal residual disease after curative treatment, monitoring of disease status during follow-up, drug selection, dynamic assessment of tumor response to treatment, detection and profiling of treatment resistance (Figure 2).

### Screening and early diagnosis

The high mortality rate of OC can be largely attributed to the difficulties encountered in early detection [23]. Thus, the majority of patients with HGSOC are diagnosed at an advanced stage where the possibility of a cure is limited [24]. As a result, the early detection of OC appears critical in reducing mortality and morbidity. In clinical practice, serum cancer antigen 125 (CA125) and ultrasonography (US) are widely employed as diagnostic tools for OC but are not useful as screening tests [25]. Last, the UK Collaborative Trial of OC Screening (UKCTOCS) trial did not demonstrate a survival benefit using US and/or CA125 assessments for the early diagnosis of OC [26, 27]. OC is relatively rare with a prevalence of approximately 1% in the population. For this reason, an effective and acceptable screening test should achieve a high sensitivity (> 75%) and very high specificity (99.6%) for early-stage disease [28].

In recent years, liquid biopsy has been investigated as a strategy for early detection of OC. The idea of exploiting liquid biopsy as a non-invasive screening tool is attractive and is based on the observation that cancer patients display higher levels of plasma cfDNA compared to healthy controls [29]. Some authors showed higher cfDNA concentration in patients with OC than in healthy women, and that the quantitative detection of cfDNA could help to discriminate malignant OC from benign ovarian disease [30–33]. While some studies have suggested that the quantitative detection of cfDNA may be more sensitive and specific than conventional tumor markers, a meta-analysis of nine studies showed an acceptable specificity (90%) but an unsatisfactory sensitivity (70%) for the diagnosis of OC [34]. This finding can be explained by the low sensitivity of cfDNA for the detection of early-stage malignancies, which are supposed to be a relevant target of screening programs.

Other studies focused on qualitative changes in ctDNA, including somatic mutations, methylation, and chromosomal aberrations that can be detected with NGS or PCR-based assays. *TP53* mutation is highly prevalent in HGSOC, accounting for more than 95% of somatic mutations [35]. Data from several studies indicate a high level of concordance between the detection of *TP53* mutation in blood and tissue samples, suggesting its potential role in the diagnosis of OC [36–39]. Calapre et al. [40] demonstrated a concordance rate varying from 60% to 100% depending on the type of NGS panel used.

Several studies have suggested the diagnostic potential of promoter methylation that leads to epigenetic inactivation of tumor suppressor genes [e.g., opioid binding protein/cell adhesion molecule-like (*OPCML*), slit homologue 2 (*SLIT2*)] as an early event during OC pathogenesis [41, 42]. Assessment of ctDNA methylation status in plasma samples from OC patients before surgery reported a significant association with abnormal methylation of tumor suppressor genes compared to healthy controls [43–45]. Widschwendter et al. [46] analyzed the methylation status of three epigenetic markers [collagen type XXIII alpha 1 chain (COL23A1), c2 calcium-dependent domain-containing protein 4D (C2CD4D), and wingless-type MMTV integration site family, member 6 (WNT6)] in the early screening setting. This analysis was able to identify OC up to two years before clinical diagnosis with a sensitivity of 58% and a specificity of 88% [46].

Chromosomal instability is another hallmark of OC, linked at least in part, by the early onset of *TP53* mutations [35]. Vanderstichele et al. [47] used WGS with low coverage to identify chromosomal instability in cfDNA and differentiated patients with OC from healthy controls with 84% sensitivity and 91% specificity.

An approach involving the analysis of body fluids other than blood might help by increasing the sensitivity of tumor DNA detection. For instance, a tumor-specific liquid biopsy as uterine cavity lavage could provide higher fractions of tumor material [48, 49].

Cohen et al. [50] analyzed circulating protein biomarkers and mutations in cfDNA using a multiomics strategy with a commercial blood test called CancerSEEK. This test used 61 amplicons for massively parallel sequencing to increase sensitivity while maintaining specificity. The sensitivity was 98% in patients with OC, but the detection rate was only 38% in the early stages [50].

Despite the potential application of liquid biopsy in the early diagnosis of OC, it’s far too early to claim its prime time in the clinic. Data currently available in this setting show highly divergent detection rates, small series of patients, significant variability in terminology, timing of sampling, and methodology of analyses, as well as in the histological subtypes of OC detected.

### Prognosis and early detection of recurrence

Although most OC patients achieve complete remission after first-line treatment, up to 70% of patients relapse within two years. CA125 and computed tomography (CT) scans are usually used during follow-up. Although CA125 is routinely applied in clinical practice as an OC biomarker, its clinical value is uncertain due to low sensitivity and specificity. In fact, half of the patients with normal CA125 have persistent disease. Additionally, CA125 might be elevated in benign conditions (e.g., endometriosis or pelvic inflammatory disease), limiting its utility in OC follow-up [51–53].

Increasing evidence from other malignancies supports the clinical application of liquid biopsy to define the risk of recurrence and its earlier detection. ctDNA analysis may serve these purposes by identifying the presence of persisting occult disease after initial treatment, likely responsible for disease relapse and poor prognosis [54–56]. Furthermore, a longitudinal collection of liquid biopsies during follow-up would identify early ctDNA changes resulting in therapeutic anticipation compared to traditional imaging-based follow-up.

In OC evidence suggested that ctDNA analysis could anticipate the detection of relapse in comparison to CA125 and radiological images such as CT and positron emission tomography (PET) scans [57–60].

Pereira et al. [57] used PCR and targeted sequencing to quantify ctDNA levels following surgery in 22 patients with HGSOC. Undetectable levels of ctDNA after adjuvant treatment were associated with significantly improved survival outcomes. The authors also showed that the detection of ctDNA anticipated the diagnosis of recurrence by approximately 7 months compared to CT imaging [57]. Similarly, in a cohort of 48 patients with HGSOC, approximately 80% of patients without residual disease after surgery had detectable ctDNA. Despite being not statistically significant, patients with detectable ctDNA had shorter overall survival [61]. Minato et al. [58] detected ctDNA in all patients (6/11) with recurrent OC using droplet digital PCR, while no ctDNA was detected in the plasma of recurrence-free patients (5/11). In 5/6 cases, the appearance of the ctDNA preceded the increase of the CA125 [58]. Paracchini et al. [59] recently used shallow WGS to calculate the percentage of tumor fraction (TF) in samples from 46 HGSOC patients. In this study, TF calculated at the time of diagnosis was an independent prognostic marker of relapse. Moreover, in longitudinal monitoring, the increase of TF preceded the CA125 by almost 8 months in the detection of disease progression [59].

These studies, although limited by their retrospective nature, small sample sizes, and lack of an independent validation cohort, suggest a potential utility of ctDNA as a predictor of OC recurrence [57–60]. Nevertheless, therapeutic options for recurrent disease are currently limited, and early detection of recurrence may not always impact survival [62]. The clinical value of an early detection of tumor relapse and the earlier start of treatment should be evaluated in well-designed prospective studies using ctDNA detection as a marker of disease recurrence.

### Assessment of response to treatment

Currently, radiological imaging remains the gold-standard to evaluate response to treatment. OC is often associated with peritoneal disease, which is usually non-measurable or hardly measurable by CT scan [63]. The serum marker CA125 has also been used to monitor treatment response, with some disadvantages, such as long half-life and poor tumor-specificity.

To date, evidence of a potential utility of serial ctDNA monitoring to predict response to anticancer treatment in OC is far preliminary. In a retrospective study of 40 patients with relapsed OC, the TP53 mutant allele frequency (TP53MAF) has been qctDNA before, during, and after chemotherapy. Pre-treatment ctDNA TP53MAF concentration correlated with initial tumor volume better than the CA125 value. Of note, patients with a > 60% decrease in TP53MAF after one cycle of chemotherapy had a longer time to progression compared to those with a decrease of less than 60% [41]. Similarly, a study showed that TP53MAF in ctDNA was significantly reduced in 28 patients with OC treated with chemotherapy, but the correlation with clinical outcomes was not reported [39]. These studies also indicate that changes in ctDNA TP53MAF could provide some indications when monitoring treatment response.

Noguchi et al. [64] retrospectively compared the tumor mutation burden in plasma samples utilizing CAPP-seq for plasma ctDNA in 10 patients with OC treated with neoadjuvant chemotherapy (6 chemo-sensitive and 4 chemo-resistant). They found that in 5 responder patients, the variant allele frequency (VAF) of non-synonymous mutations decreased after chemotherapy while it increased in 2 resistant cases, and new mutations emerged following chemotherapy [64]. In a prospective study with cfDNA samples collected from 12 HGSOC patients before, during, and after platinum-based chemotherapy, clinically actionable mutations were detected in 7 (58%) patients. Consistently with previously described studies, the authors observed that responders had a higher number of mutations with decreasing VAF when compared with poor responders. Furthermore, the authors emphasize that in a patient with platinum-resistant disease, trastuzumab was used as treatment following the detection of Erb-B2 Receptor Tyrosine Kinase 2 (*ERBB2*)amplification in ctDNA, leading to a radiological and biochemical response [65].

Rusan et al. [66] assessed the potential role as a biomarker of homeobox A9 (*HOXA9*) promoter methylation in ctDNA (meth-ctDNA) in patients with platinum-resistant *BRCA*-mutated OC, treated with a PARP inhibitor. The best clinical outcome was observed in patients with detectable *HOXA9* meth-ctDNA at baseline, but subsequent undetectable levels [66].

While further studies are necessary to define the optimal timing of sample collection and the most suitable and cost-effective assay for ctDNA measurement, serial ctDNA monitoring has the potential to complement the information available from routine imaging-based disease assessments. Identifying progression prior to the development of clinically evident disease might justify to discontinue treatments that are not providing benefit to the patients, sparing them from clinical and financial toxicity. Moreover, serial monitoring of ctDNA could be used as an early predictor of treatment efficacy to facilitate adaptive clinical trial strategies.

### Early detection and profiling of treatment resistance

The emergence of platinum-resistant disease is one of the major challenges in the management of OC.

Intratumor heterogeneity and adaptability of the OC genome under the selective pressure of chemotherapy represent one of the main reasons for drug resistance and treatment failure [67, 68]. While a “real-time” molecular characterization of recurrent disease would be crucial for intercepting actionable genetic vulnerabilities or drug resistance mechanisms, longitudinal acquisition of multiple tissue biopsies is clinically impracticable and too invasive for often heavily pre-treated patients. In this scenario, the use of liquid biopsy is a promising option to overcome this issue, allowing for monitoring of tumor evolution over time. Indeed, serial profiling of ctDNA can provide a more comprehensive picture of the tumor heterogeneity, by capturing the dynamic changes in the mutational landscape that may occur under the selective pressure of anticancer treatments [69].

In addition to blood, tumor material can also be searched in ascites [70]. In fact, many patients with recurrent OC present with malignant ascites, and it is not unusual in the clinic that large volumes need to be drained, often repeatedly, to relieve abdominal distension. Sampling ascites might allow for a more comprehensive assessment of the mutational profile at the time of each recurrence or progression [71, 72].

Nearly half of HGSOCs harbor defects in HR, which is a biomarker of platinum-based chemotherapies and PARP inhibitors sensitivity [73]. In the last decade, the introduction of PARP inhibitors has revolutionized the treatment landscape of OC in both first line and relapsed settings [7, 8, 74, 75]. However, acquired mutations that confer resistance to PARP inhibitors have been reported in some patients after long-term exposure to treatment [76, 77].

Germline or somatic pathogenic mutations in *BRCA1* or *BRCA2* are the best described mechanism of HR deficiency [78]. Accordingly, the most readily conceivable mechanism of PARP inhibitor resistance is the acquisition of reversion mutations, that restore HR function [79–81].

Recent studies have shown the feasibility to detect reversion mutations by cfDNA analysis suggesting its potential clinical use [81–83]. Christie et al. [82] conducted a prospective study in 30 patients with HGSOC carrying a germline *BRCA1/2* mutation and detected *BRCA1/2* reversion mutations in the tumor in 31.3% of patients treated in the recurrent setting, among which 18.8% also had detectable *BRCA1/2* reversions in cfDNA [82].

In a recent larger study, NGS analysis was performed on cfDNA from plasma collected prior to rucaparib treatment in 112 patients with germline or somatic *BRCA*-mutant HGSOC enrolled in the ARIEL2 trial [81]. *BRCA* reversion mutations in cfDNA were found in 18% (2/11) of platinum-refractory and 13% (5/38) of platinum-resistant patients, compared with 2% (1/48) of platinum-sensitive patients. Furthermore, patients without *BRCA* reversion mutations detected in the pre-treatment cfDNA had significantly longer progression-ree survival (PFS) on rucaparib than those with reversion mutations. In addition, the authors also sequenced 78 post-progression cfDNA samples to examine acquired resistance and identified an additional 8 patients with novel *BRCA* reversion mutations not found in pre-treatment cfDNA, suggesting the ability of cfDNA to monitor dynamic changes in *BRCA* mutational status over time [81].

Based on these studies, cfDNA sequencing was able to detect reversion mutations, but it was a relatively rare event and thus, liquid biopsy could also be used to identify unknown mechanisms of treatment resistance.

To explore the mutation profile associated with PARP inhibitor resistance, Hu et al. [84] analyzed the cfDNA pre- and post-treatment in 25 patients with platinum-sensitive HGSOC receiving maintenance with olaparib. An increased somatic mutation load in post-treatment samples was detected and it was predictive of poor prognosis. Additionally, patients with *MRE11A* (a gene involved in the DNA damage repair response) mutations in post-treatment samples had shorter PFS compared to patients without this mutation. Newly acquired mutations in *MRE11A* were associated with disease progression or resistance to ongoing treatment in more than 90% (12/13) of cases [84].

Understanding the mechanism of treatment resistance is warranted to develop novel therapeutic agents and combination, and to improve patient’s outcomes. Even though the level of evidence in OC is rather preliminary, routine monitoring through liquid biopsy could lead to earlier detection and characterization of drug resistance that may potentially create the opportunity for therapeutic interventions before clinical evident progression. Future clinical trials should implement trial designs that include longitudinal blood sampling at diagnosis, during-treatment, and at the time of each recurrence or progression.

## Current limitations for the application of liquid biopsy in OC

Despite the clinical advantages of liquid biopsy, its integration in oncology practice requires first to overcome relevant technical and biological challenges. For instance, routine adoption of liquid biopsy in clinical practice is limited by the lack of standardization in the procedures for ctDNA isolation and analysis [20]. Given the low concentration of ctDNA and its relatively short half-life, preanalytical procedures need to be well optimized in order to preserve DNA integrity [85]. Notably, it is crucial to avoid white blood cell lysis which further dilutes the amount of ctDNA. Other preanalytical variables can impact the accuracy and reproducibility of ctDNA analysis. For istance, ctDNA levels can be affected by oncological treatments or a patient’s physiological conditions such as intense physical exercise or inflammation, and these factors should be considered when selecting the timing of blood collection [85].

Another major issue to the clinical implementation of liquid biopsy is represented by the low sensitivity of many current ctDNA assays. It is well known that the ctDNA concentration is influenced by tumor stage, disease burden, and anatomical site [17, 86, 87]. False negative results might occur as a consequence of the scarce amount of target material, particularly in early-stage diseases. In this regard, ultrasensitive assays (e.g., methylation pattern-based) could be particularly suitable for the screening or the early detection of the disease since they can be performed on small amounts of ctDNA While the biological issue of DNA shedding mainly affects the sensitivity of liquid biopsy, the presence of genetic aberrations in blood originating from noncancerous cells limits the specificity of circulating DNA analyses. Clonal hematopoiesis of indeterminate potential (CHIP) occurred as a consequence of the age-related accumulation of somatic alterations in hematopoietic cells. CHIP variants are detectable in plasma DNA leading to false-positive results [88]. A study in patients with advanced-stage prostate cancer, approximately 10% of men had cfDNA harboring CHIP mutations in genes involved in DNA repair [89].

Lastly, another major limitation for the routine use of liquid biopsy in patients with OC is the lack of a prospective validation of its clinical utility. Only retrospective studies have been conducted so far (Table 1), and the results of those studies are difficult to interpret and compare mainly due to the small sample size and significant heterogeneity of the methods and outcomes. Therefore, well-designed prospective clinical trials in which ctDNA analysis results are used to inform treatment decisions and that demonstrate meaningful benefits to patients are necessary before the implementation of ctDNA assays in the clinic.

**Table 1:** An overview of main studies assessing liquid biopsy in OC

**Reference**	**Year**	**N° pts**	**Application**	**Method**	**Target**	**Sensitivity**	**Specificity**
Kamat et al. [30]	2006	19	Diagnosis	Real-time PCR	Levels of cfDNA	87–91.5%	85–87%
Capizzi et al. [33]	2008	22	Diagnosis	Real-time PCR	Levels of cfDNA	77%	96%
Shao et al. [32]	2015	36	Diagnosis	bDNA technique	Levels of cfDNA	88.9%	89.5%
Widschwendter et al. [46]	2017	43	Diagnosis	Bisulfite sequencing	Methylation in 3 markers	90.7%	41.4%
Vanderstichele et al. [47]	2017	57	Diagnosis	WGS	CNA	74%	91%
Cohen et al. [50]	2018	32	Diagnosis	CancerSEEK	Genes panel & proteins	70%	99%
Pereira et al. [57]	2015	22	Prognosis	ddPCR	Levels of cfDNA	91%	60%
Akbari et al. [61]	2019	48	Prognosis	NGS	Levels of ctDNA	NR	NR
Paracchini et al. [59]	2020	46	Prognosis	sWGS	CNA	NR	NR
Minato et al. [58]	2021	11	Prognosis	ddPCR	Levels of ctDNA	NR	NR
Parkinson et al. [41]	2016	40	Therapy response	Digital PCR	*TP53* mutation	71%	88%
Kim et al. [39]	2019	28	Therapy response	ddPCR	*TP53* mutation	NR	NR
Oikkonen et al. [65]	2019	12	Therapy response	NGS	CNV and genes panel	NR	NR
Noguchi et al. [64]	2020	10	Therapy response	CAPP-seq	CNV	NR	NR
Rusan et al. [66]	2020	32	Therapy response	ddPCR	*HOXA9* methylation	NR	NR
Christie et al. [82]	2017	30	Resistance	NGS	*BRCA1/2* reversion	60%	100%
Weigelt et al. [83]	2017	19	Resistance	NGS	*BRCA1/2* reversion	NR	NR
Lin et al. [81]	2019	112	Resistance	NGS	*BRCA1/2* reversion	NR	NR
Hu et al. [84]	2022	25	Resistance	NGS	Genes panel	NR	NR

N° pts: number of patients; bDNA: branched DNA; CAPP-seq: cancer personalized profile by deep sequencing; cfDNA: cell-free DNA; CNA: copy number alterations; CNV: copy number variations; ctDNA: circulating tumor DNA; PCR: polymerase chain reaction; ddPCR: droplet digital PCR; NGS: next-generation sequencing; sWGS: shallow whole genome sequencing; NR: not reported; HOXA9: homeobox A9; BRCA1/2: breast cancer gene 1 and 2

## Conclusions

Liquid biopsy, and in particular ctDNA analysis, is gaining increasing popularity in oncology in several clinical settings. Liquid biopsy has the theoretical advantage of being minimal invasive, repeatable over time, and able to capture spatial and temporal tumor heterogeneity. While some retrospective studies suggest a potential role of liquid biopsy to refine OC management, the published data are not consistent enough to draw any firm conclusion about its clinical applicability. Further efforts to standardize blood-based assays and incorporate liquid biopsies as a predictive biomarker in prospective trials are warranted with the goal of improving the outcome of patients with OC.

## Abbreviations

cfDNA: cell-free DNA
CNVs: copy number variations
ctDNA: circulating tumor DNA
CT: computed tomography
HGSOC: high-grade serous ovarian cancer
HR: homologous recombination
TP53MAF: TP53 mutant allele frequency
NGS: next generation sequencing
OC: ovarian cancer
PARP: poly-ADP ribose polymerase
PCR: polymerase chain reaction
WGS: whole genome sequencing
CHIP: clonal hematopoiesis of indeterminate potential
CA125: cancer antigen 125
BRCA1/2: breast cancer gene 1 and 2
TF: tumor fraction

## Declarations

### Copyright

© The Author(s) 2024.
